# Intraparenchymal intracranial pressure monitoring for hydrocephalus and cerebrospinal fluid disorders

**DOI:** 10.1007/s00701-017-3281-2

**Published:** 2017-08-10

**Authors:** Aswin Chari, Debayan Dasgupta, Alexander Smedley, Claudia Craven, Edward Dyson, Samir Matloob, Simon Thompson, Lewis Thorne, Ahmed K. Toma, Laurence Watkins

**Affiliations:** 1 Victor Horsley Department of Neurosurgery, National Hospital for Neurology and Neurosurgery, Queen Square, London, WC1N 3BG UK; 2 Division of Brain Sciences, Faculty of Medicine, Imperial College London, London, UK

**Keywords:** Intracranial pressure, Hydrocephalus, Cerebrospinal fluid dynamics

## Abstract

**Background:**

Elective intraparenchymal intracranial pressure (ICP) monitoring is useful for the diagnosis and treatment of hydrocephalus and cerebrospinal fluid (CSF) disorders. This retrospective study analyzes median ICP and pulse amplitude (PA) recordings in neurosurgically naïve patients undergoing elective ICP monitoring for suspected CSF disorders.

**Methods:**

Retrospective review of prospectively collated database of neurosurgically naïve patients undergoing elective ICP monitoring for suspected hydrocephalus and CSF disorders. Following extraction of the median ICP and PA values (separated into all, day and night time recordings), principal component analysis (PCA) was performed to identify the principal factors determining the spread of the data. Exploratory comparisons and correlations of ICP and PA values were explored, including by post hoc diagnostic groupings and age.

**Results:**

A total of 198 patients were identified in six distinct diagnostic groups (*n* = 21–47 in each).

The PCA suggested that there were two main factors accounting for the spread in the data, with 61.4% of the variance determined largely by the PA and 33.0% by the ICP recordings.

Exploratory comparisons of PA and ICP between the diagnostic groups showed significant differences between the groups. Specifically, significant differences were observed in PA between a group managed conservatively and the Chiari/syrinx, IIH, and NPH/LOVA groups and in the ICP between the conservatively managed group and high-pressure, IIH, and low-pressure groups. Correlations between ICP and PA revealed some interesting trends in the different diagnostic groups and correlations between ICP, PA, and age revealed a decreasing ICP and increasing PA with age.

**Conclusions:**

This study provides insights into hydrodynamic disturbances in different diagnostic groups of patients with CSF hydrodynamic disorders. It highlights the utility of analyzing both median PA and ICP recordings, stratified into day and night time recordings.

## Introduction

The use of elective intraparenchymal intracranial pressure (ICP) monitoring for the diagnosis and management of hydrocephalus and cerebrospinal fluid (CSF) disorders in adults has been used for several years in a number of different centers [9, 10, 20, 21]. It has multiple benefits over other diagnostic procedures such as lumbar puncture and lumbar infusion studies as it provides a longer period of monitoring, with opportunities to identify fluctuations isolated to certain periods of the day/night and track symptom-pressure correlations over a period of 24-48 h [19]. It reduces the risk of false positives and false negatives induced by ‘snapshot’ methods such as lumbar puncture. ICP monitoring also has the ability to assess response to interventions (such as CSF shunts and venous sinus stents) and has been shown to reduce the need for re-intervention in these patients [17, 18, 21]. In our experience, intraparenchymal ICP monitoring is safe, with a low rate of complications [5, 20, 21].

In our institution, elective ICP monitoring is undertaken in patients who have symptoms deemed consistent with altered CSF dynamics, following discussion at a specialist multidisciplinary team meeting consisting of neurosurgeons and neurologists with a specialist interest in headache, hydrocephalus, and CSF disorders. Following monitoring, the specialist multidisciplinary team arrives at a diagnosis, based upon a holistic assessment of the patient’s symptoms and analysis of the ICP trace over the monitoring period, the ICP histogram and the ICP vs. PA scatter plot.

Despite the benefits and relatively low risk, there are little published data on the results of invasive ICP monitoring in this context, specifically data about values to guide clinical decision-making in both naïve patients undergoing ICP monitoring and patients undergoing ICP monitoring to assess dysfunction post-interventions such CSF shunts and venous sinus stents.

This study undertakes a retrospective analysis of the median ICP and pulse amplitude (PA) recordings measured during the entire recording phase (all), day time (day), and night time (night) in this cohort of patients undergoing intraparenchymal ICP monitoring for suspected hydrocephalus and disorders of CSF dynamics. The aims of the study were to assess, via principal component analysis (PCA), which of these components are critical to the interpretation. Previous work from our center has suggested that 24-h monitoring is sufficient [19] and splitting the data into day time and night time would also allow us to assess whether overnight monitoring is required to make a diagnosis.

## Methods

A prospectively maintained database of recordings of all adult patients undergoing elective intraparenchymal ICP monitoring was retrospectively reviewed to identify all episodes of intracranial pressure monitoring undertaken at our institution between June 2006 and December 2015.

Inclusion criteria:

- Patients undergoing elective diagnostic ICP monitoring who were naïve to previous neurosurgical intervention

Exclusion criteria:

- Previous neurosurgical intervention that might have altered CSF dynamics
- Multiple episodes of ICP monitoring for that patient
- Post-ICP monitoring diagnosis unavailable from retrospective review of patient notes

All patients underwent ICP monitoring following a multidisciplinary decision. This involved the insertion of a Spiegelberg intraparenchymal ICP probe under either sedation or local anesthesia under strict aseptic conditions in an operating theatre, as part of a specialist CSF disorder neurosurgical team. Following insertion, patients were admitted to the neurosurgical ward and connected to a monitor and a computer and the data were recorded via a specialized software package, ICM+, which subtracts artefacts and records minute-by-minute data on ICP and PA. Patients were managed in a protocolized fashion, being allowed to sit up (in bed or in a chair) and mobilize during the day time and lay in bed at night. Monitoring continued for 24-48 h until the treating neurosurgical team deemed the recording to be of sufficient quality. Patients were then discharged.

**Fig. 1:**
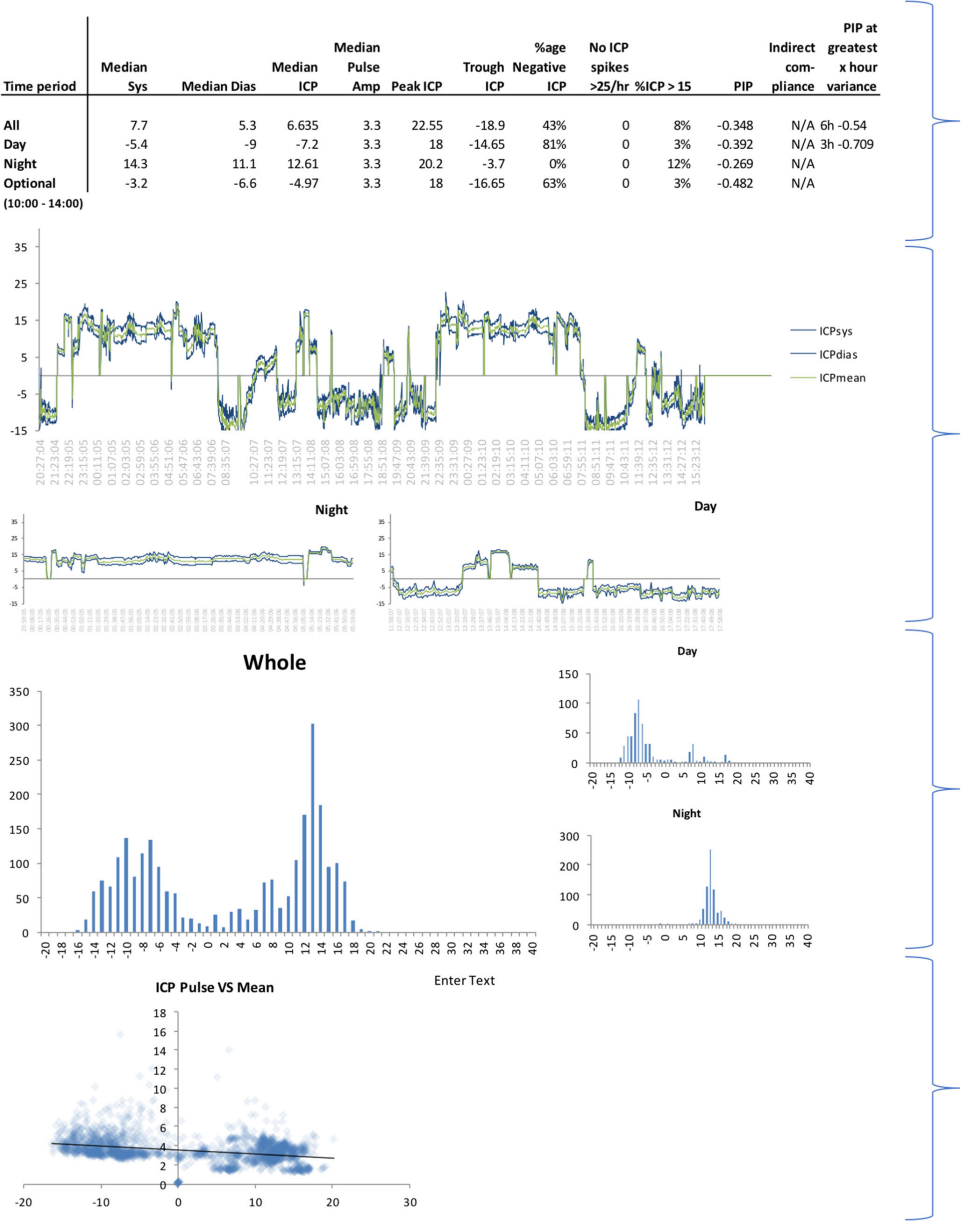
Example of in-house analysis tool used to analyze ICP recordings for each patient

Following recording, the data were analyzed using an in-house analysis tool, which isolated the median ICP and PA during the whole recording period (all), day and night times. The tool also calculated an indirect measure of compliance and had graphical representations of the distribution of ICP and a correlation between ICP and PA during the recording period (Fig. 1). The diagnosis was then determined by a specialist multidisciplinary team consisting of specialist neurosurgeons and neurologists; the team considered the clinical picture as well as the results of the ICP monitoring, including ICP trace over the monitoring period, the ICP histogram, and the ICP vs. PA scatter plot.

Following collection for this study, data were analyzed using Microsoft Excel and IBM SPSS Statistics v24. Principal component analysis (PCA) was conducted to assess the principal components of the data set. Descriptive analyses and correlations were explored to assess differences between the diagnostic groups.

## Results

**Fig. 2:**
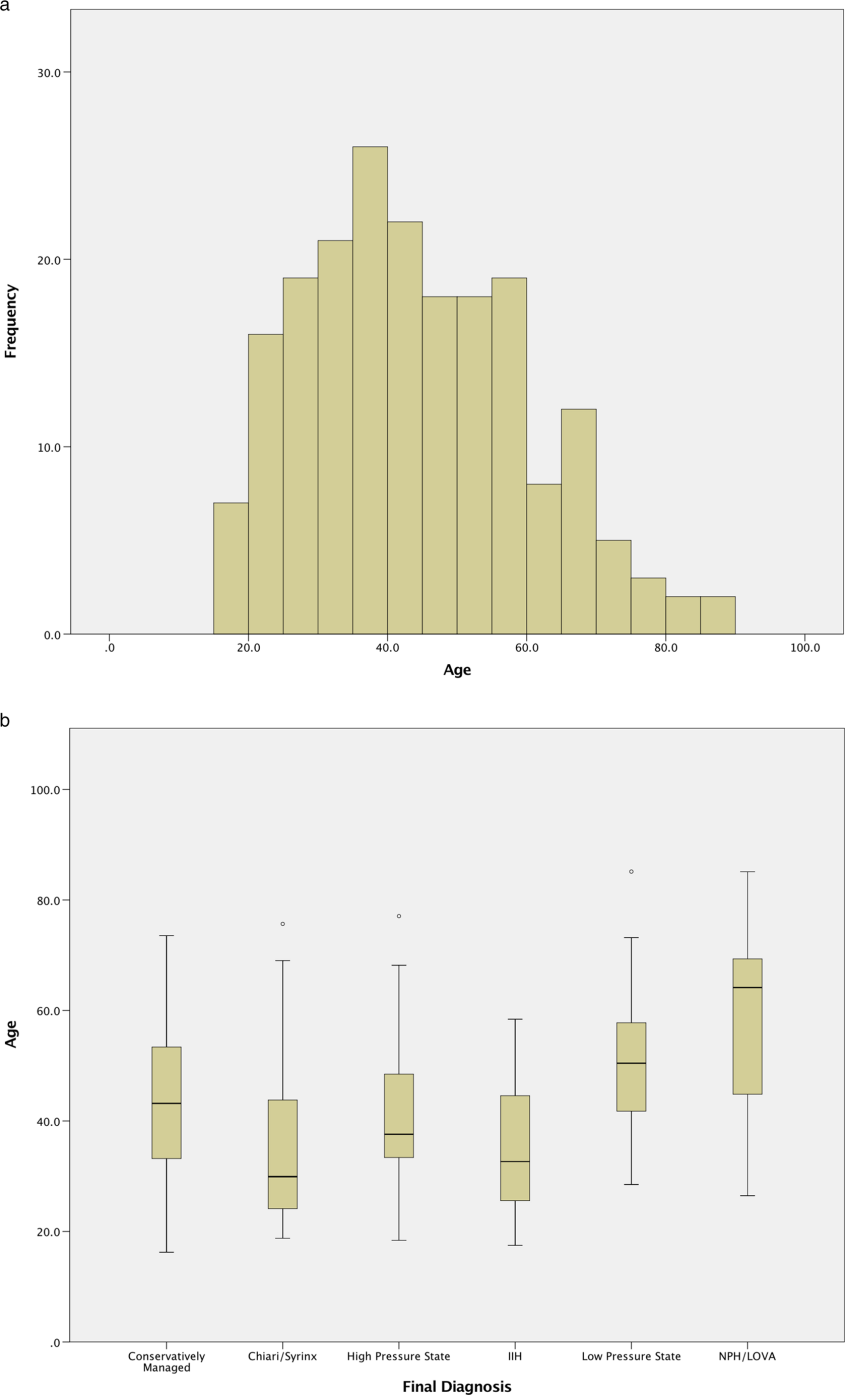
Age distribution of patients undergoing ICP monitoring. The histogram (**a**) shows the wide range of ages undergoing ICP monitoring. The box plot (**b**) shows, as expected, a younger population undergoing monitoring for Chiari/syrinx and IIH and an older population undergoing monitoring for NPH/LOVA

A total of 198 patients were identified who underwent first-time ICP monitoring over the study period who were naïve to prior neurosurgical intervention. The population demographics, split by diagnosis, are shown in Table 1. Diagnoses were split into broad categories and are described in the table. The population was diverse in terms of age, ranging from 16 to 85 (Fig. 2a), with a good range of age groups in each of the diagnostic subgroups (Fig. 2b).

**Table 1:** Diagnoses and demographic details of patients undergoing ICP monitoring

Diagnosis	*n*	Mean age	SD	Explanation of diagnosis
Conservatively managed	41	42.8	13.9	Likely to have a primary headache disorder. Further management was overseen by a specialist headache neurologist
Chiari/syrinx	21	36.0	15.8	Patients with a Chiari I malformation and/or syrinx that were deemed to be symptomatic from this
High-pressure state	37	41.3	13.5	High-pressure states included congenital, post-traumatic, post-infectious, post-hemorrhagic hydrocephalus
IIH	35	34.9	11.2	Idiopathic intracranial hypertension
Low-pressure state	37	50.8	12.7	Patients with a low-pressure state were investigated and/or had treatment for spontaneous/iatrogenic CSF leaks
NPH/LOVA	27	55.7	21.4	Normal-pressure hydrocephalus or longstanding overt ventriculomegaly in adults

### Principal component analysis

PCA was conducted on six variables (median ICP and PA, split into all, day and night) following adequate tests for sampling adequacy (Kaiser–Meyer–Olkin measure = 0.658) and sphericity (Bartlett’s test, *p* < 0.001). This analysis did not take into account the post hoc diagnostic groups. The scree plot suggested two principal components with an Eigen value >1. These normalized factors were largely determined by the three PA values (factor 1) and the three ICP values (factor 2), as shown below. Factor 1 accounted for 61.4% of the variance in the and factor 2 accounted for 33.0% of the variance in the data.

Factor 1 = 0.984 (PA (all)) + 0.956 (PA (day)) + 0.953 (PA (night)) + 0.134 (ICP (all)) + 0.137 (ICP (day)) + 0.172 (ICP (night)).

Factor 2 = 0.146 (PA (all)) + 0.133 (PA (day)) + 0.166 (PA (night)) + 0.980 (ICP (all)) + 0.949 (ICP (day)) + 0.939 (ICP (night)).

**Fig. 3:**
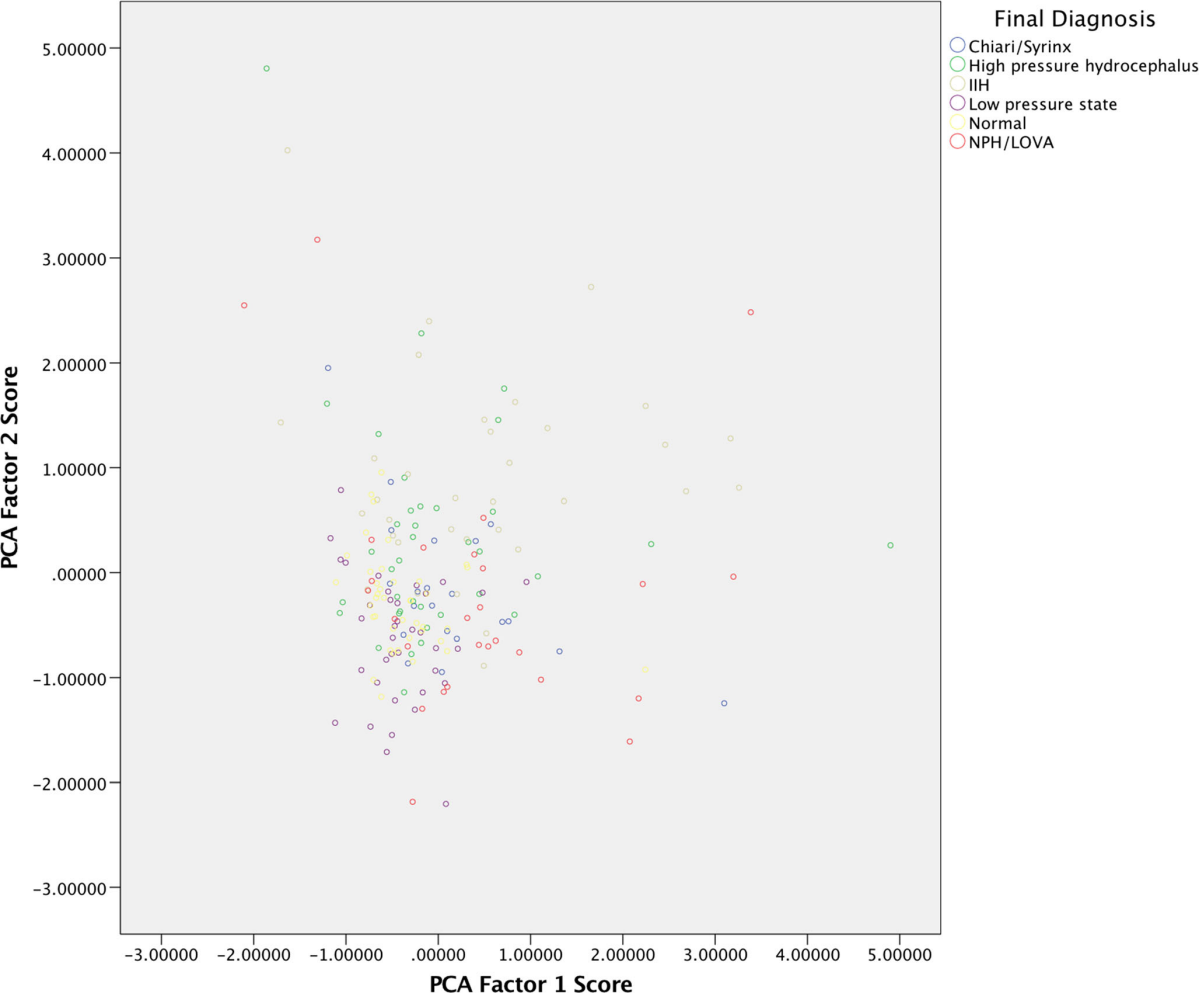
Scatter plot based on the normalized scores from factors determined by the PCA. Each* dot* represents one patient and each post hoc diagnostic is assigned a different color

A scatter plot of the factors, separated by post hoc diagnostic groups suggested separation of the diagnostic groups, with the ‘conservatively managed’ group towards the middle of the normalized data (Fig. 3).

### Descriptive analysis of diagnostic groups

The PCA suggests that all six variables play a major role in separating the data into its principal components with the PA readings constituting factor 1 and the ICP readings constituting factor 2. To explore these relationships further, a descriptive analysis of the data was carried out, based on the post hoc diagnostic groupings.

**Fig. 4:**
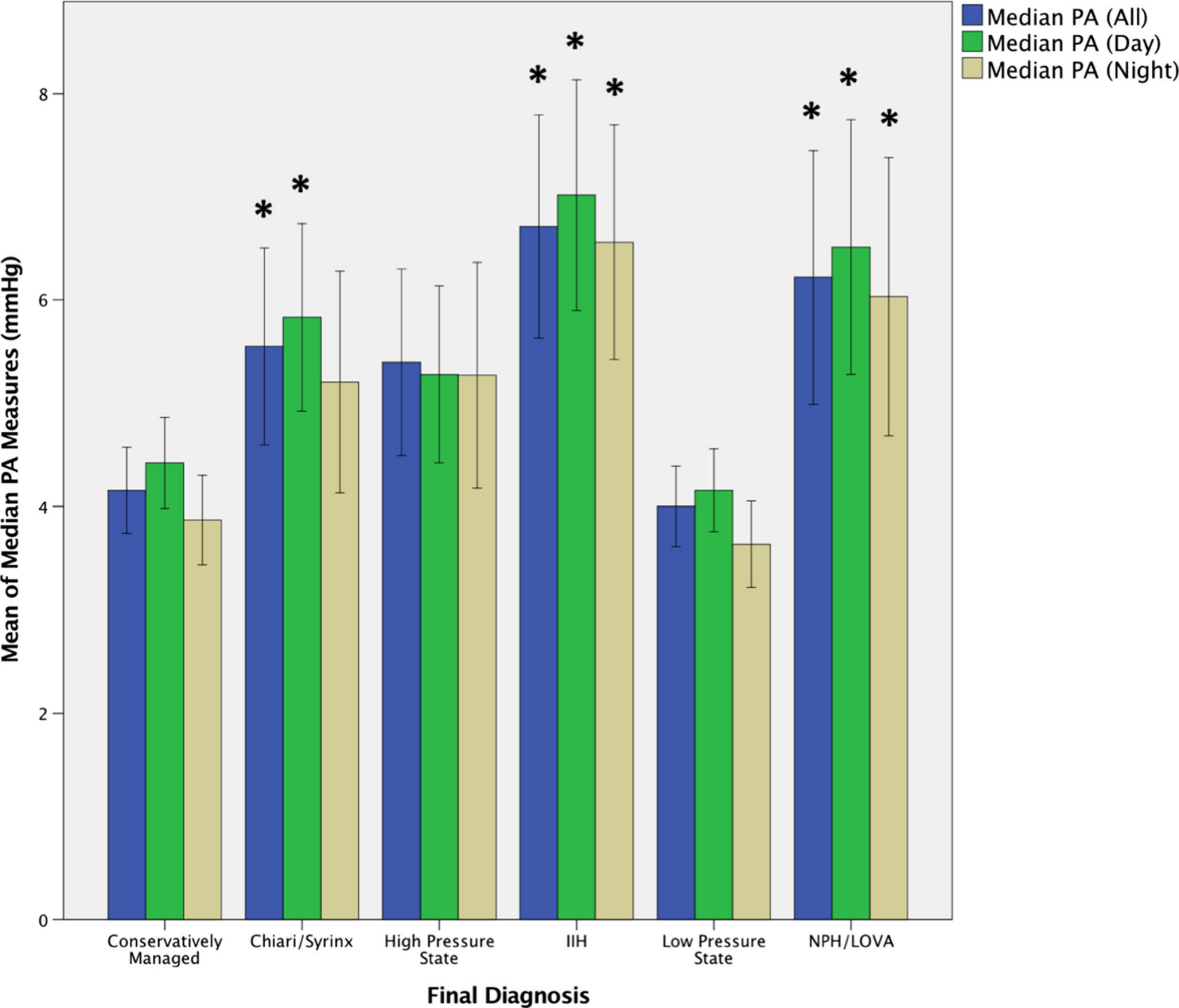
Mean recordings for median PA (all, day, and night) for the different post hoc diagnostic groups.* Error bars* denote 95% confidence intervals. * indicates a statistically significant difference between the diagnostic category and the conservatively managed group

**Fig. 5:**
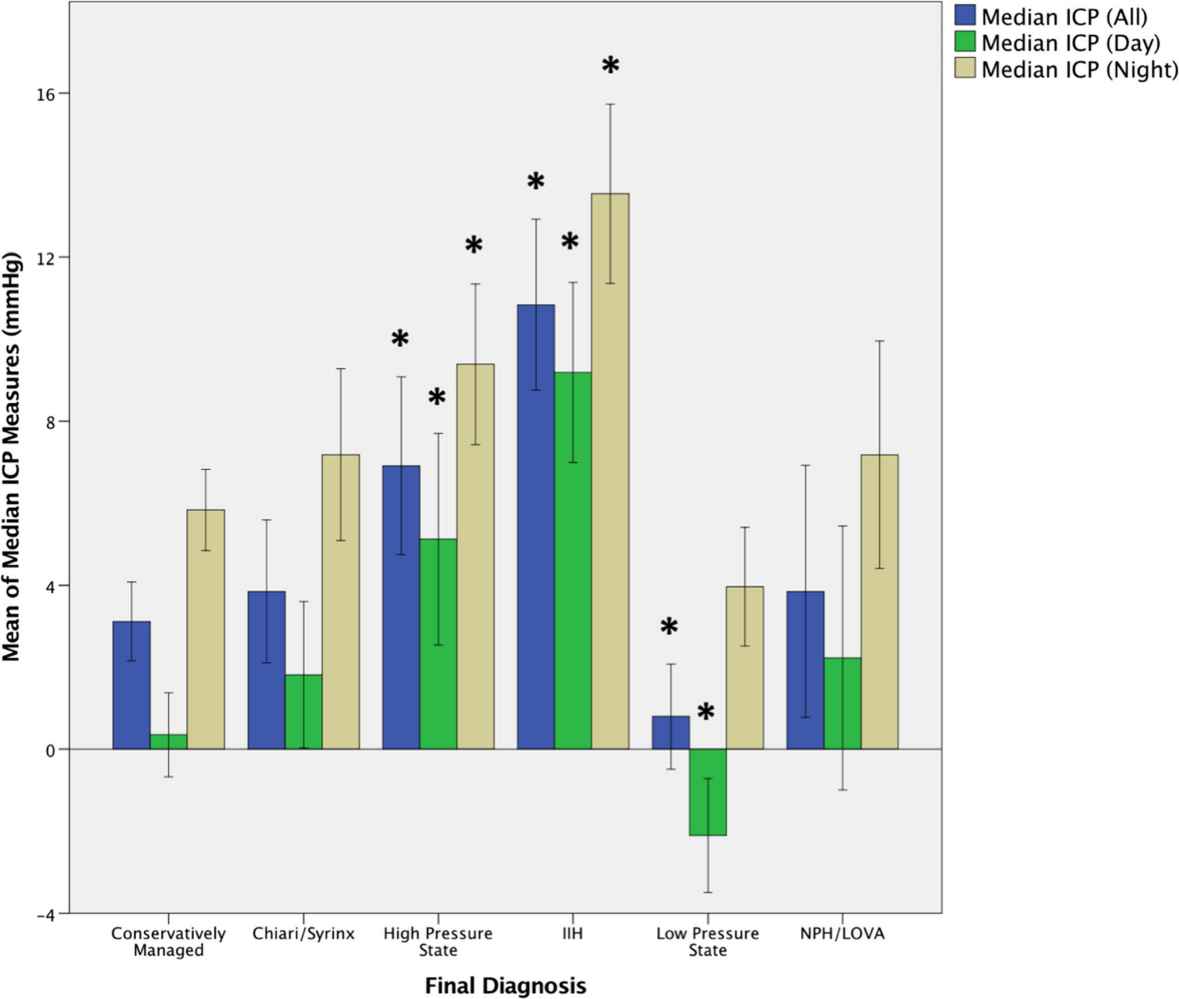
Mean recordings for median ICP (all, day, and night) for the different post hoc diagnostic groups.* Error bars* denote 95% confidence intervals. * indicates a statistically significant difference between the diagnostic category and the conservatively managed group

Mean recordings of the median PAs (all, day, and night) and median ICPs (all, day, and night) for the patients in each diagnostic group are shown in Figs. 4 and 5, respectively. Independent-sample Kruskal–Wallis test showed significant differences between the different diagnostic groups in all six paradigms. Post hoc two-way heteroscedastic* t* tests were performed with a Bonferroni correction (*p* = 0.01 as five tests were performed for each paradigm, comparing each group with the ‘conservatively managed’ group; significant results are indicated on the figures (*). These indicate median PA values were significantly different between the ‘conservatively managed’ group and the Chiari/syrinx (all and day, but not night), IIH (all, day, and night) and NPH/LOVA (all, day, and night) groups. Median ICP values were significantly different between the ‘conservatively managed’ group and groups diagnosed with IIH (all, day, and night), high-pressure states (all, day, and night) and low-pressure states (all and day, but not night).

### Correlations between ICP, PA, and age

To explore the data further, correlations were explored between ICP and PA and age. Regression curves were constructed using locally weighted scatter plot smoothing (LOESS regression) with the smoothing parameter, α, set at 90%. This method was chosen as an exploratory analysis as the relationship between the variables was not known.

**Fig. 6:**
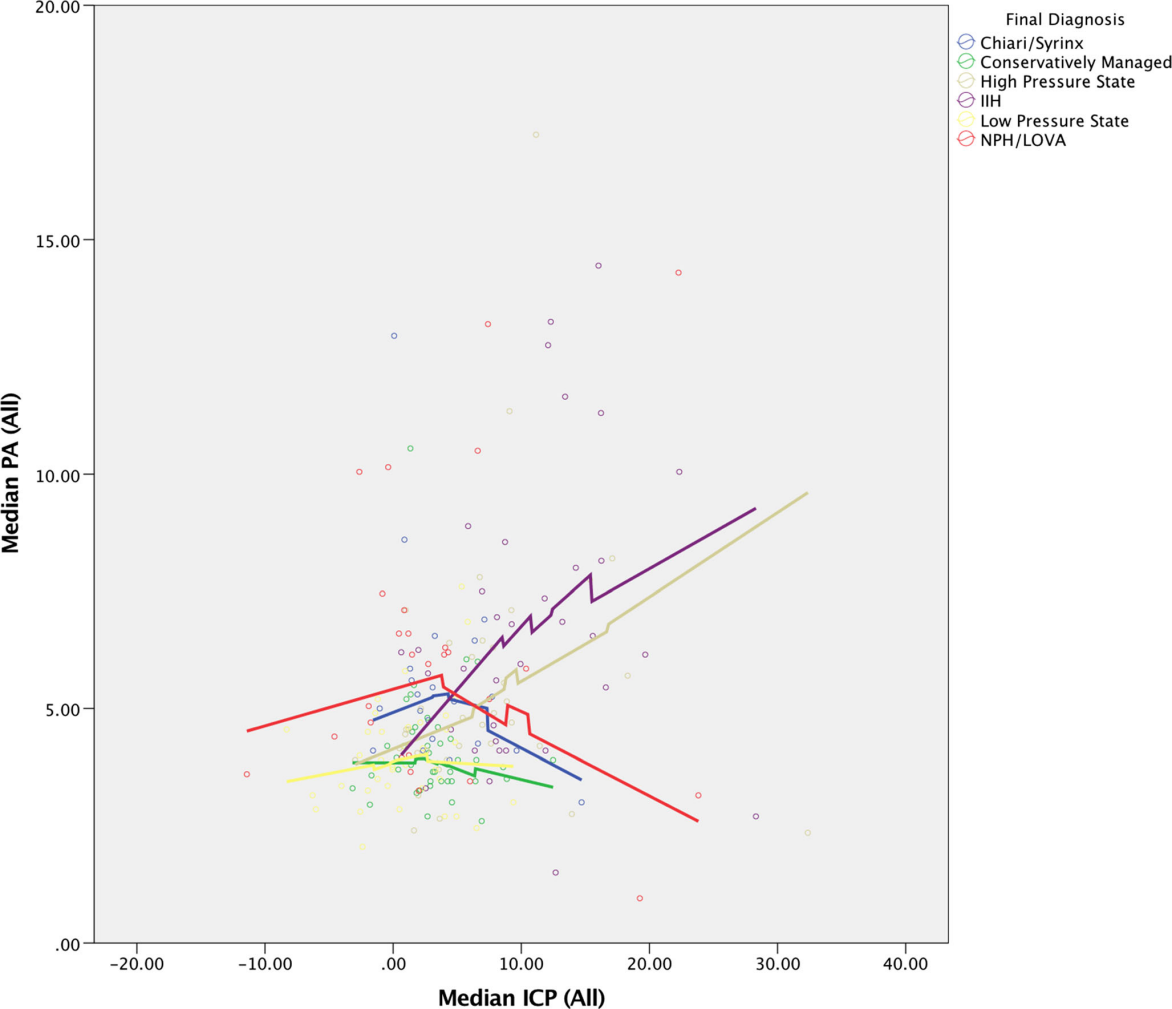
Correlation between ICP and PA, stratified by the diagnostic groups.* Lines* represent locally weighted scatter plot smoothing (LOESS) regression curves with the smoothing parameter, α, set at 90%

Assessment of the relationship between ICP and PA (Fig. 6), stratified by diagnostic subgroups, revealed a relatively flat relationship between ICP and PA for the conservatively managed and low-pressure cohorts, with a linear direct relationship for the IIH and high-pressure state cohorts. The relationship for the Chiari/syrinx and NPH/LOVA cohorts revealed an interesting trend with PA initially rising with ICP (until a median ICP of around 5 mmHg) and then decreasing, revealing perhaps a more complex physiology in these mainly compliance-associated pathologies.

**Fig. 7:**
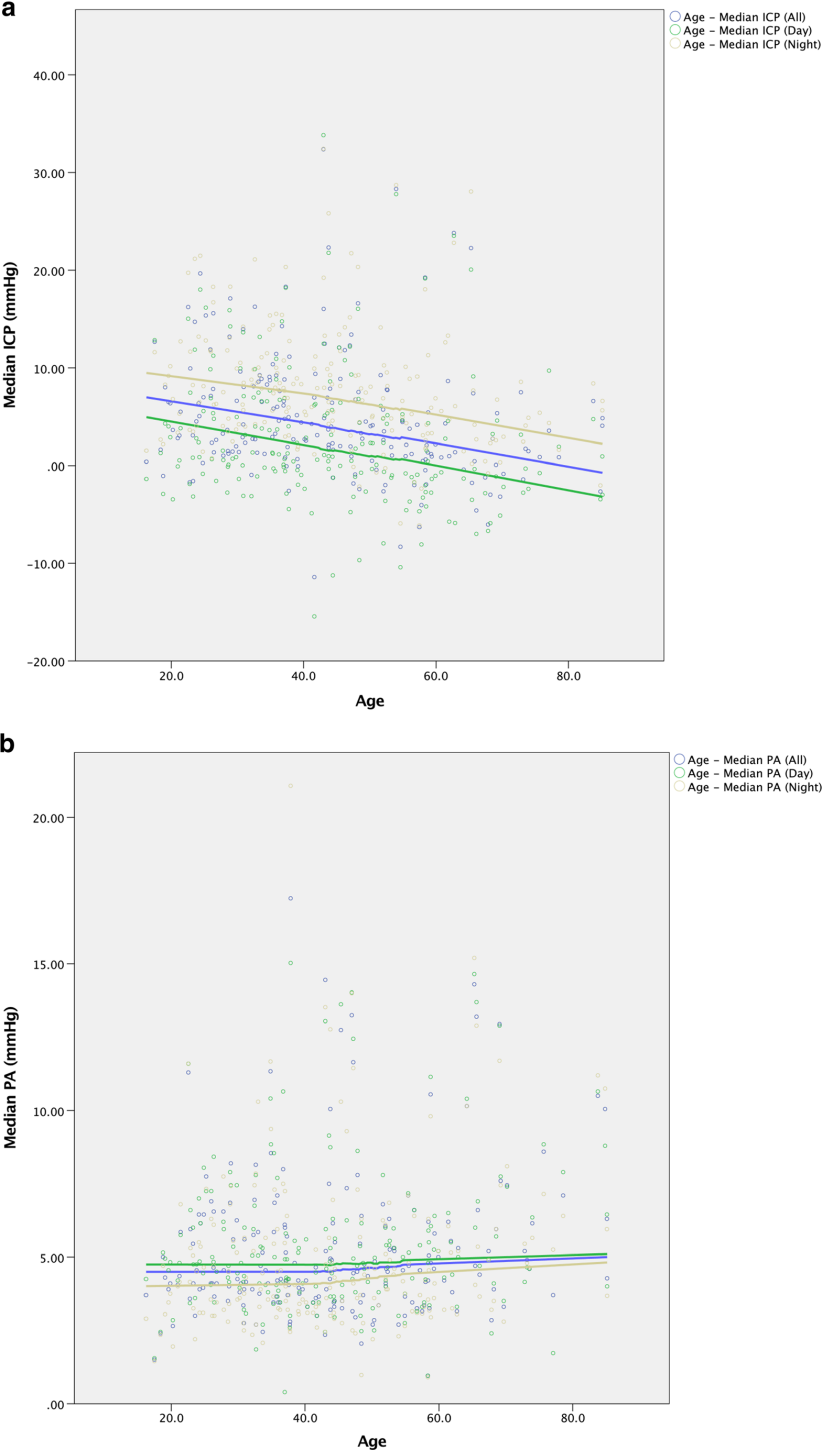
Changes in ICP (**a**) and PA (**b**) with age in the population.* Lines* represent locally weighted scatter plot smoothing (LOESS) regression curves with the smoothing parameter, α, set at 90%

Assessment of ICP and PA changes with age in the whole cohort (Fig. 7a and b respectively) revealed a slow decrease in ICP and increase in PA with increasing age, although interpretation of this is limited due to the confounds of different age distributions of the pathologies investigated (Fig. 2b).

## Discussion

We present the results of the largest series of intraparenchymal ICP monitoring in adult patients suspected of having disorders of CSF pressure and compliance who were naïve to neurosurgical intervention. The results provide valuable information about the disturbances in ICP and PA both in health and disease.

### Insight into ICP and PA in states of disease

The PCA extracted two factors with an eigenvalue >1, indicating two principal components to the data set. The first factor, incorporating all three median PA readings, accounts for almost two-thirds of the variance in the data and the second factor, incorporating all three median ICP readings, accounts for almost one-third of the variance in the data. This indicates that PA measurement might play a more important role in separating the data than ICP measurement, but that both contribute significantly to the overall spread in the data.

On exploratory descriptive analysis, significant differences were observed between the PA values in the conservatively managed group and the IIH, Chiari/syrinx and NPH/LOVA groups (Fig. 4). These findings fit with the findings of other studies that show Chiari malformations and NPH/LOVA to be primarily disorders of CSF pulsatility and compliance [8, 11–15, 20, 21]. There was a trend towards significance in the PA for the high-pressure group; it may be hypothesized that this is due to the heterogeneous nature of this diagnostic group, encompassing a number of etiologies (including post-infectious, post-hemorrhagic, and congenital), some of which may be associated with relatively normal intracranial compliance. Interestingly, the PAs were not different between the conservatively managed group and low-pressure groups, as would be predicted by the intracranial volume–pressure curves.

Significant differences were observed in the median ICP values between the conservatively managed group and those with high-pressure states, low-pressure states, and IIH (Fig. 5). Interestingly, the median ICP was no different between the low-pressure state and conservatively managed groups at night, perhaps due to redistribution of CSF associated with the supine population. There was no difference in the median ICPs between the conservatively managed and the Chiari/syrinx and NPH/LOVA groups, as would be expected from the etiologies and pathogeneses of the respective conditions.

Assessment of correlations between ICP and PA revealed some interesting trends. The differences in the regression curves between the conservatively managed cohort and the IIH and high-pressure state cohorts were expected and can be explained by a decrease in compliance in these conditions, even at low ICP values, and advocates for the measurement of PA as a marker of compliance in addition to ICP. The regression curves generated for the Chiari/syrinx and NPH/LOVA cohorts are somewhat more complex, with decreasing compliance beyond a certain ICP threshold of about 5 mmHg. This is more difficult to explain from a mechanistic perspective and perhaps accounts for the complex pathophysiology of these conditions. It remains to be seen if this relationship is true for individual patients and, importantly, in predicting treatment success in these patients.

### Insight into ICP and PA in healthy individuals

Analysis of the conservatively managed group also provides valuable insights into the physiology and dynamics of CSF in ‘normal’ individuals. Given the ethical issues surrounding monitoring ICP in normal individuals, few studies have attempted to establish the hydrodynamics of CSF in normal individuals. Previous studies have sought to establish these normal values by measuring ICP in patients following excision of small, well-demarcated brain tumors [1–3]. Although these studies have provided insight into ICP in healthy states, this is the first study to provide such data for pulse amplitudes. Although there are clear limitations with considering this pre-selected cohort of patients as entirely normal given the fact that they were chosen to undergo ICP monitoring because of their symptoms and the post hoc nature of their diagnoses, some insights may be drawn from these data.

Firstly, median ICP in this population was 3.21 mmHg (95% CI 2.29–4.13), with this being lower during the day (0.36 mmHg, 95% CI -0.62 - 1.34) and higher at night (5.84 mmHg, 95% CI 4.90–6.78), as expected by the change in posture. These values differ greatly from the accepted ‘normal range’ during a supine lumbar puncture but agree broadly with the other studies in this field [1–3]; Andresen and colleagues measure mean ICP (rather than median ICP) following removal of a small tumor and found pressures to be 0.5 ± 4.0 mmHg in the supine position and −3.7 ± 3.8 mmHg in the standing position [3]. The median pulse amplitudes also follow a similar trend, with median PA being 4.12 mmHg (95% CI 3.72–4.52) during the recordings, higher during the day (4.42 mmHg, 95% CI 4.00–4.84) and lower during the night (3.84 mmHg, 95% CI 3.43–4.25).

We also acknowledge that the recorded values are vastly different to the values and thresholds used in the sphere of traumatic brain injury (TBI) [6, 7, 16] but hypothesize that this can be explained by the chronicity of the rise in these disorders, compared to the acute rises seen in TBI.

### Insight into changes in ICP and PA with age

The changes in ICP and PA with age elucidated in this study are also of interest. The trend suggests that ICP slowly decreases with age and PA slowly increases with age. These findings must be interpreted cautiously due to the selection bias of the patients in this study. The findings could be explained by age-associated atrophy, or the fact that the age distribution for patients being investigated for conditions like IIH and high-pressure states were younger than those being investigated for conditions like NPH.

### Limitations of the study

This study has a number of limitations. It is retrospective and therefore subject to all the biases associated with retrospective studies. Perhaps the most important limitation, however, is the comparison group, designated as the conservatively managed group. These were patients chosen by a specialist multidisciplinary team to undergo ICP monitoring due to symptoms that could have been consistent with disorders of CSF dynamics; they were diagnosed as likely having a primary headache disorder post-monitoring by the same multidisciplinary team, and therefore used as a comparison group to provide insight into ‘normal’ CSF dynamics. There is clearly a circular nature to this diagnosis. However, given the ethical implications of undertaking invasive ICP monitoring in normal asymptomatic individuals, this may be the closest next-best alternative. In addition, the PCA conducted in this study was independent of the post hoc diagnostic groupings, suggesting that a composite of the median PA and ICP measures account for the variance in the data.

Another limitation is the fact that only median measures were used. This was due to the historical nature of the data and the lack of raw data for the older recordings. More in-depth analysis using minute-by-minute data for each patient is warranted. In addition, further analyses accounting for postural changes using an accelerometer and studying the within-patient correlations between ICP and PA over the monitoring period will prove invaluable in elucidating the pathophysiology of these complex disorders.

### Future directions

These findings are an important stepping stone to allow a more nuanced use of ICP monitoring for the diagnosis and management of hydrocephalus and CSF disorders. The next step would involve the development of thresholds for each diagnostic category and prospective validation of these thresholds based on the recording results, blinded to the clinical and radiological information.

Future work also needs to focus on a more detailed interrogation of the recordings, by analyzing the spread of minute-by-minute ICP and PA recordings and utilizing these detailed data to assess symptom-pressure correlations. In addition, future analyses need to build on the work of Andresen and colleagues to formulate a more detailed understanding of the relationship between posture and ICP [2]. Analyzing individual patient ICP-PA correlations may also aid the understanding of the differences between individuals, both in health and disease, with the aim of achieving optimum personalized ICP and PA targets for individuals where intracranial pressure and compliance are normalized.

## Conclusions

This study gives us insight into median ICP and PA measures in health and disease for a large cohort of patients undergoing ICP monitoring for suspected disorders of CSF dynamics. It serves as a platform for more in-depth analyses in the future and acts as a useful benchmark for the evaluation of untreated and treated patients for guided management of hydrocephalus and CSF disorders. In combination with detailed knowledge of the hydrodynamic properties of shunts [4], these data may allow us to achieve the goal of restoring ‘normal’ CSF hydrodynamics in these patients.
